# Characterization of facial nerve outcomes following radiosurgery for vestibular schwannoma: a meta-analysis

**DOI:** 10.1007/s00701-024-06405-3

**Published:** 2025-02-01

**Authors:** Gabrielle E. A. Hovis, Anubhav Chandla, Aryan Pandey, Zoe Teton, Isaac Yang

**Affiliations:** 1 Department of 1Neurosurgery, UCLA Medical Center, Harbor, Los Angeles, CA USA; 2 Departments of Radiation Oncology, UCLA Medical Center, Harbor, Los Angeles, CA USA; 3 Departments of Head and Neck Surgery, UCLA Medical Center, Harbor, Los Angeles, CA USA; 4 Departments of Jonsson Comprehensive Cancer Center, Harbor-UCLA Medical Center, Los Angeles, CA USA; 5 Los Angeles Biomedical Research Institute, Harbor-UCLA Medical Center, Los Angeles, CA USA; 6 Harbor-UCLA Medical Center, Los Angeles, CA USA

**Keywords:** Vestibular schwannoma, Gamma knife, Radiosurgery, Facial nerve preservation

## Abstract

**Purpose:**

Gamma Knife radiosurgery (GKRS) is a precise and efficacious treatment modality for vestibular schwannoma (VS) with favorable cranial nerve preservation rates. This study aims to better characterize facial nerve (FN) outcomes in VS after GKRS.

**Methods:**

A query of six medical databases was conducted following PRISMA guidelines. Eligible studies exclusively reported VS managed with single-fraction GKRS and included House-Brackmann (HB) scale assessments prior to and following GKRS. Data was analyzed using random-effects modeling, and FN preservation was defined as HB I or II at last follow-up.

**Results:**

Data was analyzed from 15 articles with 3,155 patients at an mean age of 55.0 years. Mean tumor volume, radiation dose, follow-up, tumor control, and hearing preservation were 4.28 cm^3^, 13.3 Gy, 59.4 months, 92.7%, and 62.6%, respectively. The pooled FN preservation rate was 92.9%. Mean preoperative tumor volume > 2.5 cm^3^ and age > 60 years were significantly associated with worse preoperative FN function (*p* = 0.019, *p* = 0.023, respectively). Normal FN function (HB = 1) at last follow up was 95.8% for VS volume < 2.5 cm^3^ and 89.4% with larger volumes (*p* < 0.001). Doses ≤ 13 Gy were significantly associated with superior FN preservation (96.5%) compared to higher doses (*p* < 0.001). Tumor control and hearing preservation were not significantly associated with FN preservation.

**Conclusion:**

This meta-analysis identifies tumor volume and radiation dose as prognostic factors for FN preservation. A FN preservation rate of 93% may be expected at five years after GKRS. This study provides a unique characterization of FN outcome that should be considered in the management of VS.

## Introduction

Vestibular schwannoma (VS), previously termed acoustic neuroma, is a benign, slow-growing tumor with an estimated incidence of 1:100,000 [22, 82]. VS is the most common neoplasm of the cerebellopontine angle and comprises approximately 6–7% of intracranial tumors [44, 47, 50, 56, 82].

There is a lack of high-quality evidence identifying an optimal treatment strategy for VS. Current treatment options include observation, stereotactic radiosurgery (SRS), and microsurgical resection. Observation is a reasonable treatment plan for patients with incidental, asymptomatic VS,^22,21^ but may result in inferior tumor control and hearing preservation rates relative to SRS [7, 22, 30, 80, 99]. In general, SRS is recommended for small and medium VS (diameter < 3 cm, Koos grade I and II), while resection is preferred for large, symptomatic VS (diameter ≥ 3 cm, Koos grade III and IV) [22, 71, 74]. For large VS, subtotal resection with adjuvant SRS has demonstrated superior facial nerve (FN) and hearing preservation rates relative to gross total resection. [13, 22, 36] .

One form of SRS is the Gamma Knife technique, developed by Swedish neurosurgeon Lars Leksell in 1968 [45, 63]. The technology uses Cobalt-60 as a radiation source to provide a precise, non-invasive approach to tumor treatment. The safety and efficacy of Gamma Knife radiosurgery (GKRS) for the treatment of VS is well documented, particularly for neoplasms less than 3 cm in diameter. [5, 6, 9, 24, 26, 27] .

While tumor control remains the primary objective in VS management, improved techniques have made patient-centered outcomes increasingly attainable [3, 86]. Cranial nerve (CN) VII function is a primary determinant of patient quality of life in VS management [46, 58, 59, 76]. While the incidence of facial neuropathy following GKRS is well supported, there are few studies investigating prognostic factors for FN preservation in VS after GKRS. Most published studies are limited by smaller sample sizes, retrospective study designs, single-institution data, and potential physician or institutional bias. This study seeks to better characterize FN preservation following GKRS for VS by conducting a comprehensive meta-analysis of the existing literature.

## Methods

### Data collection

Full-text articles in the English-language literature were identified according to the Preferred Reporting Items for Systematic Reviews and Meta-Analyses (PRISMA) guidelines. [26] .

**Fig. 1:**
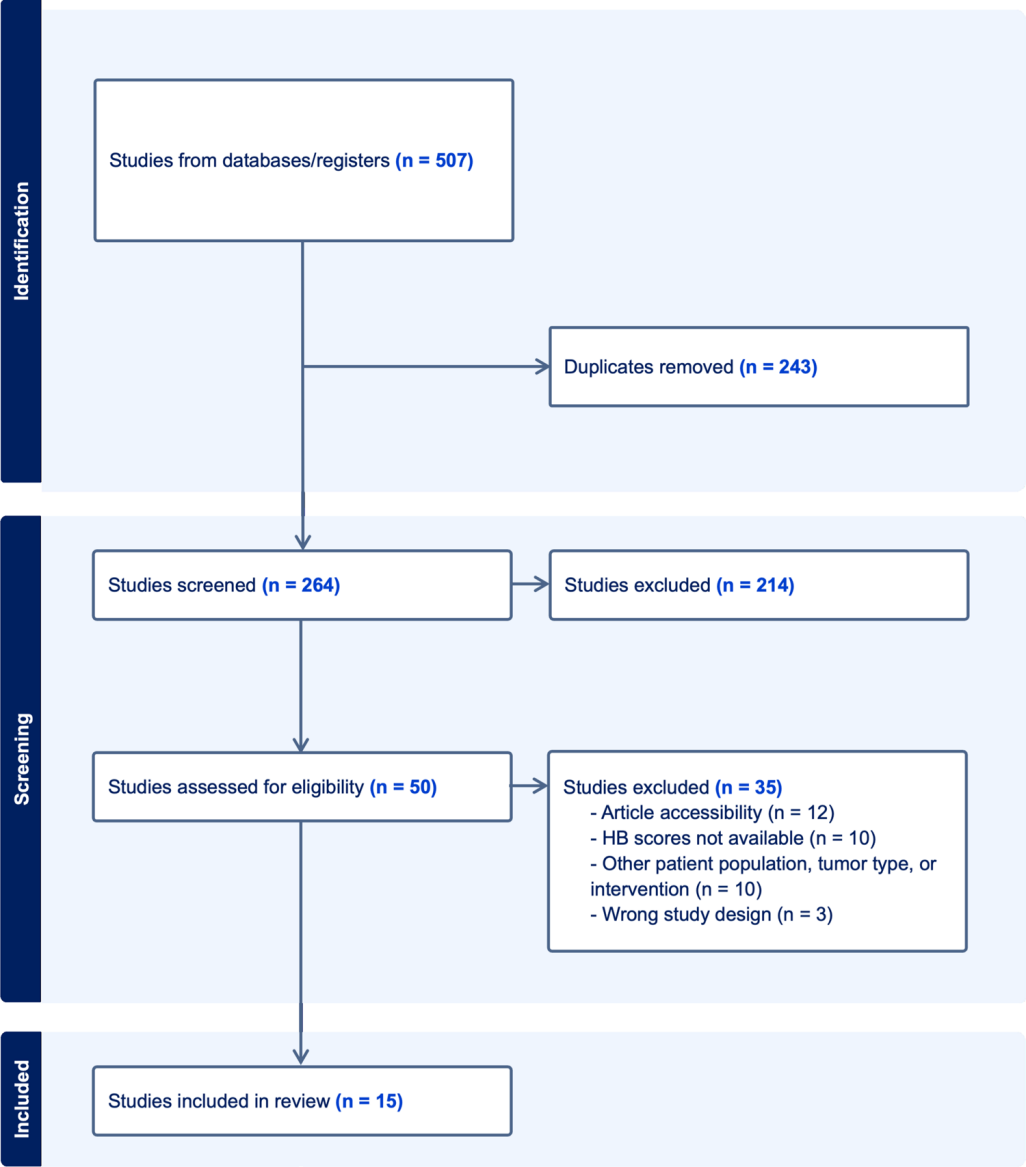
Preferred Reporting Items for Systematic Reviews and Meta-Analyses (PRISMA) flow diagram with details of study selection

Five databases (PubMed, Web of Science, Scopus, Cochrane, Embase, and MEDLINE) were queried for articles up to and including the year 2023 using the following search terms alone and in combination: “gamma knife,” “vestibular schwannoma,” “acoustic neuroma,” “house-brackmann.” Using the Covidence systematic review software [12], two reviewers independently screened literature for the following criteria: 1) VS was the sole tumor target, 2) GKRS was the only form of radiosurgery used for treatment, 3) House-Brackmann (HB) scale [32] was reported prior to and following GKRS, and 4) radiotherapy was delivered via a single-fraction regimen. Article selection according to PRISMA guidelines is shown in Fig. 1. Studies consisting of pediatric populations, pregnant patients, conference abstracts, book chapters, reviews, case reports, inaccessible full-texts and non-English texts were excluded. Patients with neurofibromatosis type II (NF2) and those who underwent adjuvant GKRS or radiosurgery other than GKRS were also excluded.

### Data extraction

Demographic data was collected per individual study, and postoperative HB scores ≤ II were tabulated. Normal FN function was defined as an HB score of I, and FN preservation was characterized by HB ≤ II at the last reported follow-up visit. The mean follow-up time was used for this calculation. Tumor volume, marginal radiation dose, presenting symptoms, postoperative complications, tumor control rate, and hearing preservation rates were noted.

### Statistical analysis

Available data for each study was analyzed using random-effects modeling to account for heterogeneity and interstudy variation. Descriptive statistics were reported as pooled values to control for effect size. FN function was quantified using the House-Brackmann grading system, [32] and the Gardner-Robertson (GR) scale [20] was used to assess hearing ability.

A meta-analysis was performed for all variables of interest using a significance threshold of p ≤ 0.05. Pre- and postoperative HB scores were compared with clinical outcomes using subgroup meta-analysis to verify the efficacy of GKRS. Values reported as medians were converted to means using the *estmeansd* package in RStudio [84] and associated web-based application. [8, 51, 52] .

In an included comparison study of large and small VS tumors, Williams et al. [32] reported data separately for each of the cohorts. This data was analyzed as two individual datasets due to heterogeneous demographics and baseline CN function between the groups.

## Results

A total of 15 articles with 3,155 patients were included in our analysis. The mean age was 55.0 (range 29–62) years, with a mean follow-up period of 59.4 (range 24.0–107) months. Patient demographics for each study can be found in Table 1.

**Table 1:** Patient and tumor characteristics and facial nerve function among the selected GKRS series

**Series (first author, year)**	Patients (N)	Age, *Mean*	Marginal Dose, *Mean (Gy)*	Tumor Volume, *Mean (cm*^*3*^*)*	Preoperative HB I + II, N (%)	Postoperative HB I, N (%)	Postoperative HB I + II, N (%)
Boari 2014 [27]	379	59.0	13.0	1.20	376 (99.2)	368 (97.1)	–-
Ogunrinde 1994 [70]	98	51.7	17.6	3.20	82 (83.7)	61 (62.2)	73 (74.5)
Yang 2011 [94]	65	53.0	12.0	9.00	55 (84.6)	65 (100)	64 (98.5)
Frischer 2019 [18]	452	55.3	12.0	1.20	445 (98.5)	447 (98.9)	–-
Kondziolka 1998 [43]	162	60.0	16.6	–-	135 (83.3)	122 (75.3)	–-
Litvack 2003 [48]	134	55.3	12.0	–-	–-	126 (94.0)	–-
Hempel 2006 [29]	123	54.3	13.0	1.60	121 (98.4)	111 (90.2)	121 (98.4)
Myrseth 2009 [76]	60	57.5	12.0	–-	60 (100)	59 (98.3)	59 (98.3)
Lobato-Polo 2009 [49]	55	35.0	13.0	0.002	55 (100)	54 (98.2)	54 (98.2)
Park 2011 [72]	31	59.7	14.2	–-	29 (93.5)	26 (83.9)	29 (93.5)
Zeiler 2013 [100]	25	56.0	12.5	–-	–-	19 (76.0)	–-
Williams 2013 [92]**	24	61.5	11.0	9.52	20 (83.3)	16 (66.7)	20 (83.3)
Williams 2013 [92]**	49	61.8	12.0	0.70	43 (87.8)	33 (67.3)	36 (73.5)
Johnson 2019 [37]	871	57.0	13.0	0.98	–-	816 (93.7)	–-
Pikis 2023 [71]	627	54.0*	12.0*	8.70*	604 (96.3)	576 (91.9)	604 (96.3)

**Data reported as median**

^**^Distinct cohorts from the same study

*GKRS*, gamma knife radiosurgery *HB*, House-Brackmann grade

### Presenting signs/symptoms

Prior to GKRS, 2,025 (64.2%) of patients had a HB score of I or II. Of the reported presenting symptoms, pooled analysis revealed 82.9% of patients with hearing loss, 43.5% with tinnitus, 31.6% with vertigo or disequilibrium, 29.6% with ataxia, 12.3% with trigeminal neuropathy (facial numbness or neuralgia), 12.2% with headache, and 3.0% with dysphagia. Hydrocephalus was present in 0.1% of patients prior to GKRS.

### Facial nerve function

Pooled analysis revealed an overall FN preservation rate (HB I or II) of 92.9% at the last follow-up visit. A postoperative decrease of one or more HB grades relative to preoperative HB was recorded in 1.8%. New or worsening hemifacial spasm was seen postoperatively in 2.43%. VS volume ≥ 2.5 cm^3^ and age ≥ 60 years were significantly associated with worse preoperative FN function (*p* = 0.019, *p* < 0.0001, respectively). Poor preoperative CN VII function was significantly correlated with reduced FN preservation rate postoperatively (*p* = 0.019).

### Tumor volume

**Fig. 2:**
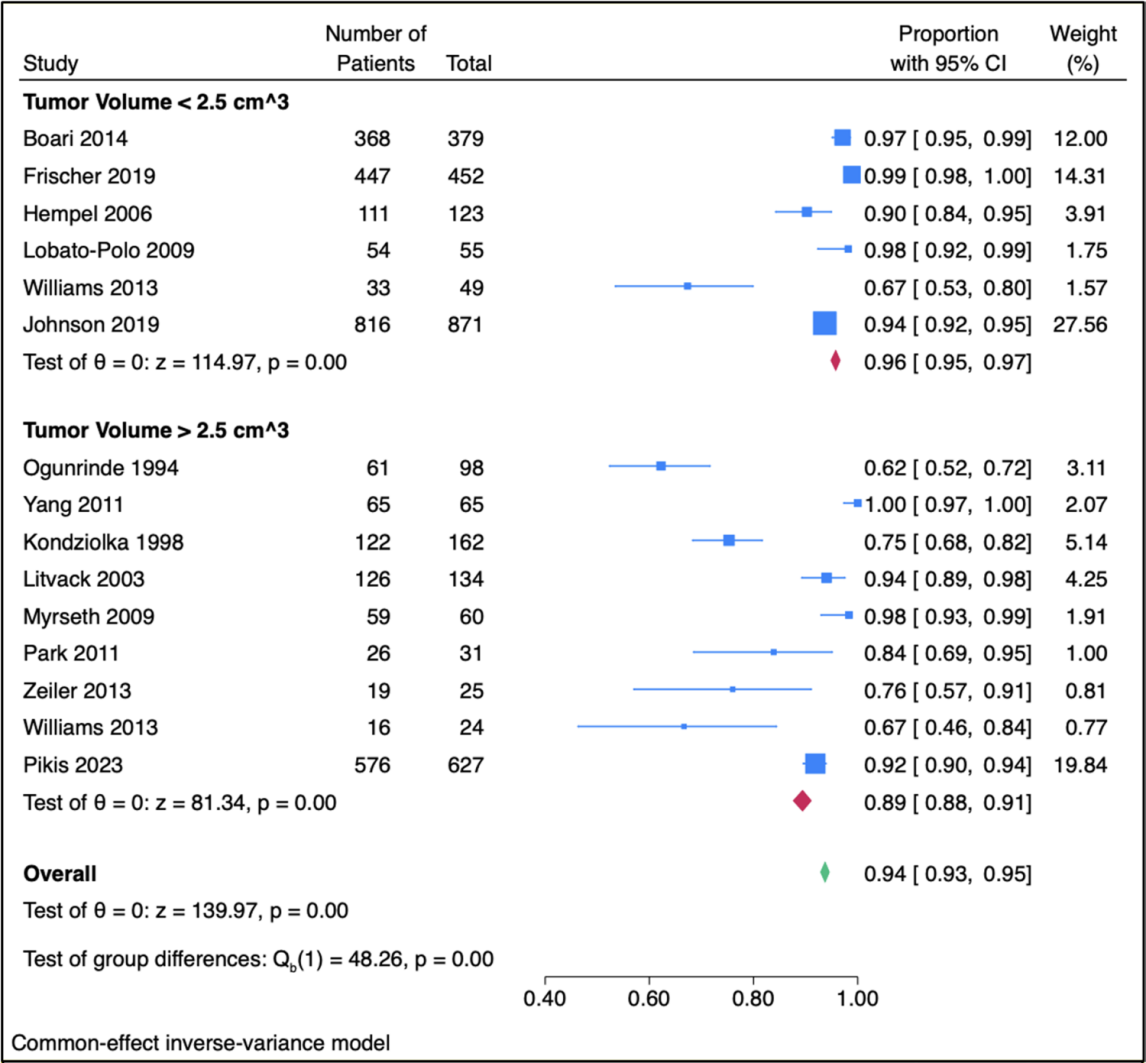
Normal postoperative facial nerve function (House-Brackmann grade I) in patients with tumor volumes less than and greater than 2.5 cm.^3^. 95.8% of patients with small- to medium-sized VS prior to radiosurgery maintained normal FN function (HB = I) at the last follow-up visit, relative to 89.4% with large VS (*p* < 0.001). Tumor volume was not significantly associated with FN preservation (HB I or II)

Postoperatively, 95.8% of patients with VS < 2.5 cm [3] in volume maintained normal FN function (HB = I) at the last follow-up visit, relative to 89.4% with VS volumes ≥ 2.5 cm^3^ (*p* < 0.001, Fig. 2). Tumor volume was similar between patients with preoperative and postoperative HB scores of I or II (normal to mild dysfunction).

### Radiation

**Fig. 3:**
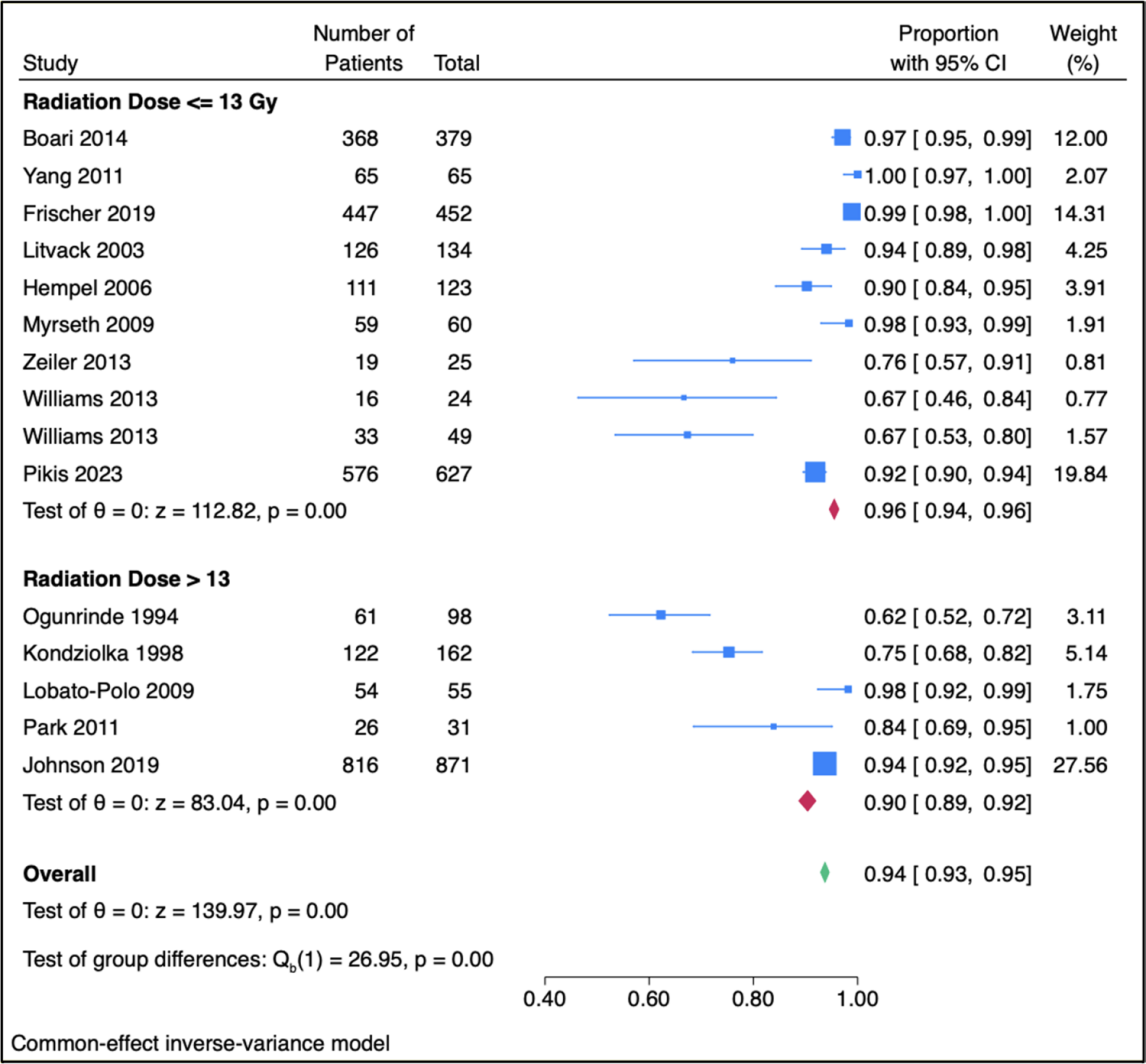
Facial nerve preservation rates with marginal radiation doses of 13 Gy or less and greater than 13 Gy. Marginal radiation doses ≤ 13 Gy were significantly associated with superior FN preservation (95.5%) compared to doses above 13 Gy (*p* < 0.001)

Normal FN function was maintained postoperatively in 91.8% with doses ≤ 13 Gy and 84.8% with doses > 13 Gy (*p* = 0.286). Marginal radiation doses ≤ 13 Gy were significantly associated with superior FN preservation (95.5%) compared to doses above 13 Gy (90.4%, *p* < 0.001, Fig. 3).

### Patient age

Age was not significantly correlated with normal postoperative FN function or FN preservation (*p*= 0.238, *p*= 371, respectively). Normal postoperative FN function was similar between age ≥ 55 years (88.6%) and age < 55 years (91.6%, *p* = 0.664). Facial nerve preservation was 94.7% for age ≥ 55 years and 94.5% for age < 55 years (*p* = 0.324).

### Tumor control and complications

The mean tumor control rate was 92.7% at a mean follow-up period of 59.4 months. The pooled values of reported complications following GKRS included need for additional surgical resection (2%), need for additional SRS (1%), and shunt placement for new or worsening hydrocephalus (2.5%). Tumor control was not significantly associated with FN preservation. Tumor control and complication rates for each study can be found in Table 2.

**Table 2:** Tumor control, postoperative CN deficits, and complications among the selected GKRS series

**Series (first author, year)**	Patients (N)	Tumor Control Rate (%)	FN Toxicity*, N (%)	Hearing Preservation (%)	New TNO, N (%)	HCP with Shunt, N (%)	Post-GKRS Resection, N (%)	Repeat GKRS, N (%)
Boari 2014 [27]	379	97.1	4 (1.1)	49	21 (5.5)	16 (4.2)	8 (2.1)	3 (0.8)
Ogunrinde 1994 [70]	98	97.0	21 (21.4)	–-	–-	–-	3 (3.1)	–-
Yang 2011 [94]	65	87.0	1 (1.5)	82	4 (6.2)	4 (6.2)	7 (10.8)	1 (1.5)
Frischer 2019 [18]	452	92.0	1 (0.2)	34	–-	13 (2.9)	6 (1.3)	–-
Kondziolka 1998 [43]	162	94.0	3 (1.9)	47	23 (14.2)	–-	4 (2.5)	–-
Litvack 2003 [48]	134	96.7	0 (0)	62	6 (4.5)	4 (3.0)	3 (2.2)	–-
Hempel 2006 [29]	123	96.7	0 (0)	82	7 (5.7)	3 (2.4)	–-	4 (3.3)
Myrseth 2009 [76]	60	–-	1 (1.7)	68	–-	2 (3.3)	1 (1.7)	–-
Lobato-Polo 2009 [49]	55	96.0	1 (1.8)	87	4 (7.3)	–-	–-	2 (3.6)
Park 2011 [72]	31	97.0	1 (3.2)	45	–-	–-	–-	–-
Zeiler 2013 [100]	25	92.0	0 (0)	100	–-	4 (16.0)	1 (4.0)	–-
Williams 2013 [92]**	24	81.2	2 (8.3)	–-	–-	2 (8.3)	3 (12.5)	3 (12.5)
Williams 2013 [92]**	49	90.0	1 (2.0)	–-	–-	0 (0)	0 (0)	0 (0)
Johnson 2019 [37]	871	94.0	14 (1.6)	51	51 (5.9)	15 (1.7)	11 (1.3)	6 (0.69)
Pikis 2023 [71]	627	87.6	19 (3.0)	–-	48 (7.7)	7 (1.1)	18 (2.9)	1 (0.16)

^*^Postoperative decrease in HB score by ≥ 1 grade

^**^Distinct cohorts from the same study

*CN*, cranial nerve *GKRS*, gamma knife radiosurgery *FN*, facial nerve *TNO*, trigeminal neuropathy *HCP*, hydrocephalus

### Hearing and trigeminal nerve function

Preoperative serviceable hearing (GR I or II) had a pooled rate of 37.4%. The pooled hearing preservation rate was 59.6%, with a mean audiometric follow up of 78.8 months.

Trigeminal neuropathy was defined as a temporary or permanent change in facial sensation (hypoesthesia or neuralgia) in the distribution of the trigeminal nerve. The pooled rate of new postoperative trigeminal neuropathy was 6.7%.

### Study bias assessment

**Fig. 4:**
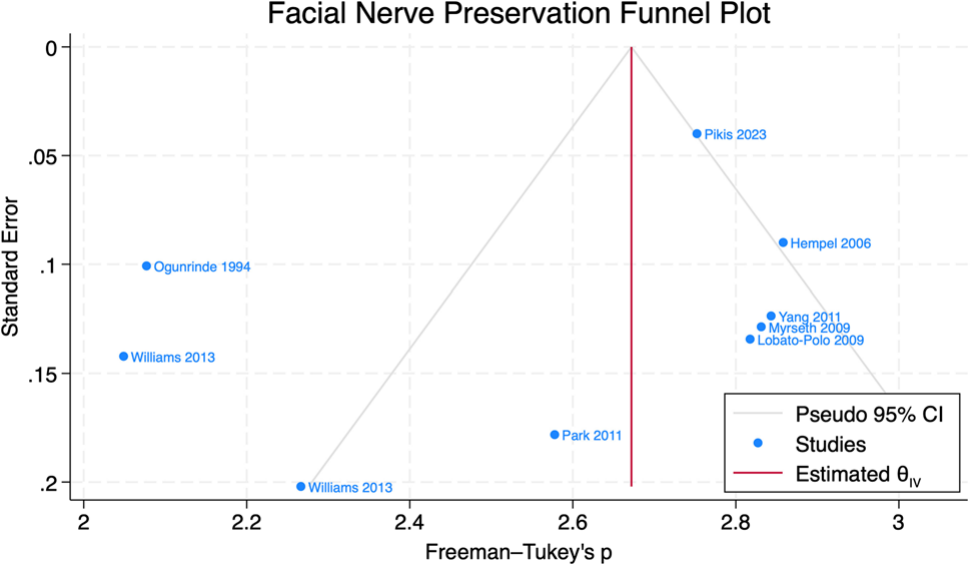
Funnel plot assessing bias in selected studies for facial nerve preservation. The grey oblique lines indicate 95% confidence boundaries of study variation, and each point represents a study of interest. Deviation of points from the aggregate Freeman-Tukey transformed proportion (vertical red line) indicates systematic bias. No significant publication bias was identified by Egger’s test for facial nerve preservation (*p* = 0.38)

Publication bias was assessed using funnel plot asymmetry and Egger’s test using a random-effects model. No significant study bias was identified by Egger’s test for FN preservation (*p* = 0.38) across all reviewed studies (Fig. 4).

## Discussion

Facial nerve preservation is paramount in the treatment of vestibular schwannoma due to its profound impact on quality of life [46, 58, 59, 76]. In its infancy, the rate of FN impairment in GKRS ranged from 30–40% [6, 65]. However, with the refinement of GKRS techniques, FN dysfunction has diminished to less than 2% [6, 10, 15, 16, 26, 65, 89, 93]. In our meta-analysis, pooled overall FN preservation was 92.9%, with a mean FN toxicity of 1.80%. This is supported by a review of 1,908 VS patients by Yang et al., who found an overall FN preservation rate of 96.2% following GKRS [95].

GKRS has also demonstrated superior FN outcomes relative to other treatment modalities for VS. Rates of CN VII impairment have been found to be higher after microsurgery relative to GKRS for small- to medium-sized VS (maximum diameter < 3 cm) in both prospective and retrospective studies with similar tumor sizes between groups [2, 23, 59, 75, 76]. Furthermore, in a meta-analysis of 1,409 VS patients, Balossier et al. found a significantly lower FN deficit among patients undergoing GKRS compared to single-fraction linear accelerator (LINAC) treatment [4]. Favorable cranial nerve preservation rates after GKRS relative to other interventions highlights the value of optimizing FN outcomes in gamma knife therapy for VS.

A multitude of factors influence facial nerve preservation in GKRS. In the present study, we found that preoperative HB scores were significantly correlated with FN function at the last follow-up visit, which is supported by prior literature. [6] This suggests that HB scores are important to consider in preoperative evaluation and selection of optimal management strategies. As such, GKRS may be more beneficial for patients with absent or mild deficits of CN VII on presentation.

The relationship between increased tumor volume and FN dysfunction following GKRS is well documented [65, 70, 92, 95]. However, gamma knife therapy has also shown favorable FN outcomes for large VS, with a FN preservation rate ranging from 90–100% [11, 33, 55, 90, 94, 98, 100]. As only two studies included mean tumor volumes above 10 cm [56],^92,94^ we were unable to evaluate the relationship between large tumor volume and FN outcomes according to standard tumor size classifications [33, 53, 66, 98]. However, we found a significant correlation between tumor volume and preoperative FN function, which in consistent with increased brainstem compression and FN involvement in larger VS [14, 79, 81]. The results also demonstrate that VS volumes < 2.5 cm^3^ had superior long-term postoperative CN VII function relative to volumes ≥ 2.5 cm^3^, though the vast majority of these patients retained their FN function (93.5% mean postoperative HB I or II). These findings illustrate that while tumor volume should be considered in management decisions and the associated moderately increased risk profile should be discussed when counseling patients, GKRS should certainly remain an option for most VS tumor sizes.

Our comprehensive analysis revealed that higher facial nerve preservation is more likely with a marginal radiation dose of 13 Gy or less compared to doses above 13 Gy. This is consistent with prior literature demonstrating that radiation dose is predictive of CN toxicity (including FN) after GKRS [17, 31, 60, 61, 73, 85, 95]. High doses of gamma knife radiation were used in the initial stages of radiosurgery, but the use of lower doses in recent years has resulted in fewer complications with preserved tumor control rates. [16, 43, 67, 76, 77] .

Increased age has been shown in some studies to pose a greater risk of FN impairment after GKRS [28, 67, 95]. In a retrospective analysis of 383 patients undergoing GKRS for VS, Kawashima et al. similarly found that age was not significantly associated with CN deficits [39]. This is reflected in the present study and suggests that other factors, such as tumor volume or radiation dose, may be stronger predictors of FN outcome.

Five-year tumor control rates in VS after GKRS range from 59.7—99.0% [6, 9, 19, 27, 57, 58, 77, 80, 88]. This observed variation is indicative of the lack of uniformity in the definition of tumor control across studies in the VS literature. Tumor control failure following GKRS is frequently reported as either the need for further treatment (surgical resection, additional SRS, other) or radiological tumor progression, while other definitions are unclear or not reported [9, 16, 42, 54, 60, 88, 91]. In 2016, Klijn et al. addressed these disparate definitions and demonstrated that these differences can significantly impact reported rates [42]. We did not account for variation in the definition of tumor control in this study which may have affected the pooled value. However, our pooled tumor control rate of 92.7% at a mean follow-up of nearly five years is consistent with the 5-year tumor control rate reported in a number of large studies with varying definitions of tumor control [19, 26, 42, 88]. Notably, in a prospective study comparing microsurgical resection to GKRS in VS patients, Pollock et al. found no significant difference in tumor control rates between the two groups with a slightly shorter mean follow-up period of 42 months. [76] .

A minority of patients in this study had serviceable hearing preoperatively (pooled 37%). However, approximately 60% had preserved hearing at their last reported audiometric follow-up (mean 6.6 years). This value is comparable to rates of 50–60% reported in other meta-analyses [4, 97]. In addition, this pooled value reflects the reported serviceable hearing preservation rate in VS over the last decade, which ranged from 27–64% at 10 years following GKRS [1, 37, 38, 68, 69]. The wide range of hearing preservation following GKRS suggests that preoperative hearing function influences hearing preservation, which has been shown in prior studies [6, 40, 41, 67]. However, variation in hearing preservation rates is multifactorial and prospective randomized trials are needed to assess this observation.

The pooled rate of new trigeminal neuropathy was 6.7% following GKRS. This value is relatively higher than previous large reports of trigeminal impairment following GKRS for VS. Sughrue et al. recorded new trigeminal neuropathy at a rate of 2.3% in a meta-analysis of 5631 patients, which increased with a marginal radiation dose above 13 Gy. [87] However, our rate of new trigeminal impairment is consistent with rates ranging from 0—14% reported in the literature after GKRS for VS. [6, 11, 34, 35, 55, 78, 83, 94] .

Most, if not all, of the included studies reflect the standard practices of academic medical centers. These standards, which already demonstrate variation across academic centers, may have greater variation among community medical centers. A comprehensive elucidation of the prognostic factors for outcome of VS patients undergoing GKRS is an essential step in establishing standardized practices for radiosurgery in VS across all institutions. [96] .

### Limitations

As a meta-analysis, this study has inherent limitations. Most of the studies in this analysis were retrospective, with the exception of one 2009 prospective, non-randomized clinical trial [58]. Due to the nature of aggregated analysis, there was a lack of uniformity across the included studies in sample size, patient demographics, and reporting outcomes. FN preservation is inconsistently reported, with unique characterization across series. Typically, it is reported as either the proportion of patients with maintenance of the same HB score or maintenance of HB scores of I and/or II postoperatively. To account for this discrepancy, we estimated the FN preservation rate for each study using HB scores ≤ II at the last follow-up visit. Even so, this variation significantly limited our sample size. While all studies reported HB scores of I postoperatively, six papers did not specify the number of patients with both HB I and II. As single-fraction GKRS is a well-established and long-standing treatment modality for VS, we selected for only articles with single-fraction GKRS with an aim to design a more uniform analysis with an emphasis on long-term outcomes. However, there are a multitude of radiosurgery modalities and fractions which may be used in VS management, and we are currently conducting a new study to compare the efficacy of different SRS modalities. Follow-up time varied between reports, and the number of patients lost to follow-up was not specifically reported for each study. Treatment failure of VS is typically identified within three years, and the literature demonstrates that the minimum follow-up period should be at least five years [25, 43, 62, 64]. The mean follow-up period for our cohort neared this threshold at 4.9 years. Nevertheless, the studies included in our analysis with a follow-up period of less than five years may have skewed the outcome results. We controlled for overall interstudy variability using pooled analysis and subgroup meta-analysis, which account for effect size and heterogeneity. However, future prospective, randomized studies are needed to verify the correlations identified in our analysis.

## Conclusion

This study represents the most comprehensive meta-analysis on facial nerve preservation following isolated gamma knife radiosurgery for vestibular schwannoma to date. Our results demonstrate an overall FN preservation rate of 93% at a mean of five years following GKRS and identify preoperative HB, tumor volume, and radiation dose as prognostic factors for facial nerve preservation. These findings offer utility for practitioners in the development of individualized management strategies to optimize facial nerve preservation.

## Data Availability

Not applicable.

## Abbreviations

CN: Cranial nerve
FN: Facial nerve
GKRS: Gamma knife radiosurgery
HB: House-Brackmann
LINAC: Linear accelerator
PRISMA: Preferred Reporting Items for Systematic Reviews and Meta-Analyses
NF2: Neurofibromatosis type II
SRS: Stereotactic radiosurgery
VS: Vestibular schwannoma
